# Immunohistochemical Evaluation of Fibronectin and Tenascin Following Direct Pulp Capping with Mineral Trioxide Aggregate, Platelet-Rich Plasma and Propolis in Dogs’ Teeth 

**DOI:** 10.7508/iej.2015.03.009

**Published:** 2015-07-01

**Authors:** Saeed Moradi, Nasrollah Saghravanian, Siavash Moushekhian, Samar Fatemi, Maryam Forghani

**Affiliations:** a *Dental Materials Research Center and Department of Endodontics, Dental School, Mashhad University of Medical Sciences, Mashhad, Iran; *; b *Oral and Maxillofacial Disease Research Center and Department of Oral and Maxillofacial Pathology, Dental School, Mashhad University of Medical Sciences, Mashhad, Iran;*; c * Private Endodontist; *; d * Dental Research Center and Department of Endodontics, Dental School, Mashhad University of Medical Sciences, Mashhad, Iran*

**Keywords:** Fibronectin, Immunohistochemistry, Propolis, Pulp Capping, Tenascin

## Abstract

**Introduction:**

The aim of the present study was to evaluate the expression of fibronectin (FN) and tenascin (TN) after direct pulp capping (DPC) in dogs’ teeth with either mineral trioxide aggregate (MTA), Propolis or Platelet-rich plasma (PRP), by means of immunohistochemistry.

**Methods and Materials:**

A total of 48 sound molars and premolars with mature apices from four dogs, were included. The teeth were randomly divided into 4 groups according to the material used for DPC: PRP, Propolis, MTA, and glass-ionomer (as the negative control group). Each group was divided into two 7-day and 30-day subgroups. The teeth were restored at the same session. The animals were sacrificed at the mentioned time intervals and the expression of FN and TN in each test group and between each time intervals was assessed with Wilcoxon and Mann-Whitney U tests, respectively. The Kruskal-Wallis test was used to compare FN and TN staining among the test groups. The significance level was set at 0.05.

**Results:**

The amount of FN in the MTA group in the 30-day interval was significantly higher than the 7-day interval; however, there were no significant differences among the other groups. The amount of TN in the MTA and Propolis groups in the 30-day interval was significantly higher than that in the 7-day interval; no recognizable difference was observed in the other groups. Moreover, the difference in expression of FN and TN in the 7-day interval was not significant in the experimental groups. Nevertheless, the difference was significant in the 30-day interval, with the highest and lowest expressions belonging to the MTA and glass-ionomer groups, respectively.

**Conclusion:**

Based on the results of the present animal study, MTA is still a better choice for direct pulp capping

## Introduction

Preservation of pulp vitality is one of the aims of endodontic treatment. There are several vital pulp therapy (VPT) techniques including pulp capping and pulpotomy [1, 2]. Various materials have been employed as pulp capping agents [3, 4]. Traditionally, several compositions containing calcium hydroxide, have been used [5] and in recent years, MTA has been suggested as far as direct pulp capping (DPC) is concerned [6]. As the pulp response to DPC materials, hard tissue barrier (HTB) is formed beneath the capping agent for which the recruitment and proliferation of undifferentiated cells to form secondary odontoblasts is held responsible [7]. Although MTA has been successful in inducing the formation of HTB [8], it entails certain disadvantages such as poor handling characteristics, delayed setting time, tooth discoloration and high cost [9]. 

Accordingly, a variety of materials have recently been proposed as candidates for DPC, such as Propolis and Platelet-rich Plasma (PRP). Propolis (*aka *bee glue) is a resin, produced by honeybees that has been used for centuries in traditional medicine as an anti-inflammatory and antibacterial agent. Of all the constituents of Propolis, flavonoids ensue different effects such as regulating the immune response, decreasing the release of free radicals and preventing the growth of bacteria and fungi [10, 11]. DPC using Propolis resulted in HTB comparable to that with MTA [12]. 

PRP is a rich source of growth factors (GFs) used in different branches of dentistry, including maxillofacial surgery, oral surgery and periodontology; it is now under investigation for VPT [13]. It has been demonstrated that PRP has good tissue compatibility and has hard tissue induction abilities [14].

The components of pulpal extra-cellular matrix (ECM) can induce reactionary and reparative dentin formation [7]. Fibronectin (FN) and tenascin (TN) are two non-collagenous glycoproteins of the ECM, expressed during dentinogenesis. Both molecules are concentrated in the dental basal membrane and can induce differentiation of odontoblasts [15]. FN contains adhesive molecules with high molecular weight and has several isoforms. This glycoprotein has an important role in migration, adhesion, proliferation and differentiation of cells [16]. TN is a large oligomeric glycoprotein in the ECM, expressed during the growth and maturation of teeth [17]. TN is important for the differentiation of odontoblasts, so it might be associated with secondary dentin formation [18]. Expression of TN increases in the presence of GFs such as transforming growth factor-*α* (TGF-*α*) and also mechanical stresses [17]. 

There is no reported immunohistochemical study focusing on DPC using PRP or Propolis. Therefore, the aim of the present study was to evaluate the expression of FN and TN following DPC with MTA, Propolis and PRP at 7- and 30-day intervals using immunohistochemistry in dogs’ teeth.

## Materials and Methods

This interventional animal study was carried out in the Animal Research Center of Mashhad Faculty of Dentistry, Mashhad, Iran. The study protocol was approved by the Ethics Committee and the Research Council of Mashhad University of Medical Sciences (ID 910043). Four healthy dogs with 64 apically mature teeth including 28 molars and 36 premolars with no cracks and periodontal problems were included in the study. Pre-operative periapical radiographs were taken under general anesthesia. The teeth were randomly divided into four groups (*n*=16) with a statistically randomized treatment table: PRP, Propolis, MTA and light-cured glass-ionomer (LCGI) (as the negative control group); each group was then again divided into 7-day and 30-day subgroups (*n*=8). 

Half an hour before the procedures, 1.0 mL of intramuscular diazepam (Chimidarou, Tehran, Iran) was injected for sedation, followed by the intramuscular injection of 10 mg/kg of anesthetic agent ketamine HCL (Rotex Medica, Germany) and 1 mg/kg of zylazine (Rotex Medica, Germany). 

After induction of the general anesthesia, 10-mL blood samples were taken from the animals. Next, citrate was added to blood samples at a ratio of 1:9 to prevent clot formation. The samples were then sent for the preparation of PRP. 

The surface of each tooth was cleaned with pumice paste (Kemdent, Swindon,Wiltshire, UK) and the teeth were isolated with rubber dam. Diamond #14 fissure burs (Jota AG, Rüthi, SG, Switzerland) installed in a high-speed handpiece were used to prepare access cavities; pulpal exposure points were located in the central pit of the teeth. A piece of sterile cotton pellet impregnated with 5.25% NaOCl was placed on the exposure point for hemostasis. 

Each tooth received the pulp cap material according to its relevant group. Propolis was provided by the beehives in Hezar Masjed Mountains and was processed in the Razi Institute Research Center in Mashhad Iran. Each mL of water-based Propolis contains 7-10 mg of the effective material (polyphenol). The material was prepared in jelly consistency to facilitate its placement and the teeth were then restored with LCGI (Fuji II LC, GC Corporation, Tokyo, Japan). In MTA group, ProRoot MTA (Dentsply, Tulsa Dental, Tulsa, OK, USA) was mixed according to the user’s manual and placed on the exposure site. In PRP group, the jelly PRP was injected into the cavities up to the level of the cementoenamel junction and allowed to clot. After DPC in all teeth sterile parafilm was placed between the DPC material and the restoration and all cavities were restored with LCGI. 

Tow animals were sacrificed after each time interval (7 and 30 days). The teeth were extracted and fixed in 10% formalin for 10 days, followed by immersion in normal saline for one day. Next, for decalcification, the teeth were placed in an agitator containing 17% ethylenediaminetetraacetic acid (EDTA) (Asia Chemi Teb. Co., Tehran, Iran) for 6 months. During this time, EDTA was refreshed every other day. After decalcification, samples were embedded in paraffin. Subsequently, 4 µm-thick sections were prepared that were used for immunohistochemical staining of FN and TN [19]. The sections were fixed on poly-L-Lysine-coated glass slides, deparaffinized and rehydrated. Slides were incubated for 30 min in 3% hydrogen peroxide/methanol and then irrigated with phosphate-buffered saline (PBS) for 20 min. For antigen retrieval, the sections were microwaved for 35 min in citrate solution (0.01 M, pH 6.0). 

**Figure 1:**
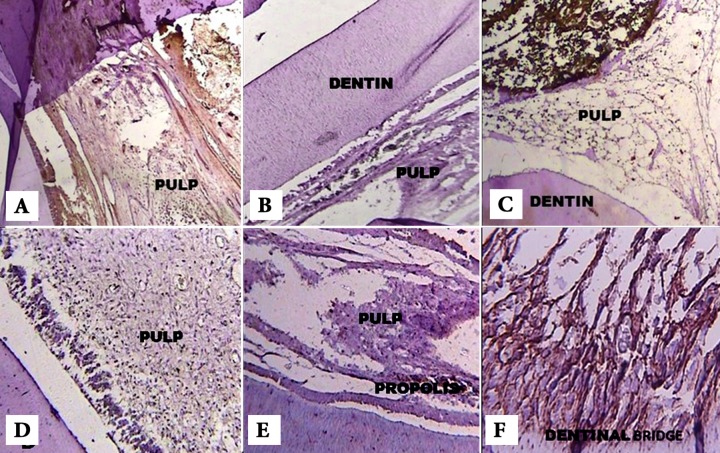
*A)* Expression of FN (grade *III*) in the PRP group after 7 days (100× magnification), *B)* Expression of FN (grade *II*) in the Propolis group after 7 days (100× magnification), *C)* Expression of FN (grade *I*) in the MTA sample after 7 days (100× magnification), *D)* Expression of TN (grade *II*) in the PRP group after 30 days(×100 magnification), *E)* Expression of TN (grade *II*) in the Propolis group after 30 days (100× magnification) and *F)* Expression of TN (grade *IV*) in the MTA group after 30 days (400× magnification

Specimens were incubated with the primary antibodies for 1 h at room temperature and then rinsed for three times with PBS. The secondary antibody was applied and immune complexes were identified by streptavidin peroxidase (NovoLink Polymer detection system, Novocastra Laboratories Ltd., Newcastle Upon Tyne, UK). After washing with PBS for three times, the immune reactivity was visualized by 3, 3’-Diamino-benzidine and hydrogen peroxide. Finally, slides were counterstained with Hematoxylin and cover-slipped with a synthetic mounting media. Lyophilized mouse monoclonal antibody (Fibronectin, NCL-FIB, IgG1 and Tenascin C, NCL-TENAS-C, IgG2b) (NovoLink Polymer detection system, Novocastra Laboratories Ltd., Newcastle Upon Tyne, UK) were used according to the manufacturer’s instructions. The stained slides were evaluated under a light microscope (Leica DME, Leica Microsystems Inc., Buffalo, New York, USA) at 40×, 100× and 400× magnification (Figure 1). 

The staining intensity of each marker was evaluated in a blind manner and was graded based on the following scale: *scale* *I*- no staining; *scale II*- weak staining (light brown); *scale III*- medium staining (oak brown); and *scale IV*- severe staining (dark brown). After determining the staining intensity of FN and TN in each sample, the results were put to statistical analyses. The Wilcoxon test was used to compare TN and FN staining in each test group and Mann-Whitney U test was employed to compare FN and TN staining between 7- and 30-day intervals. The Kruskal-Wallis test was used to compare FN and TN staining among the test groups.

## Results

From the 64 included teeth, 16 teeth were damaged during section preparation and discarded from the study. Finally, 12 teeth in each group remained for evaluations.

***Histological findings***

In 7-day samples, the pulps capped with MTA, Propolis, and PRP showed fibrous tissue formation. In MTA and Propolis samples, certain diffused calcified areas were also observed. Negative control samples (capped with GI) showed scattered inflammatory cells in the pulp; a few diffused fibrous tissues were observed, as well. 

In 30-day samples, a calcified HTB was formed under both MTA and Propolis. In PRP samples, formation of a fibrous matrix with calcified areas could be observed. Sever inflammatory infiltrate was the dominant feature of the GI samples.

***Immunohistochemical findings***

Expression of FN in the MTA group in 30-day samples was significantly higher than the 7-day specimen (*P*=0.014). In

PRP, Propolis and GI groups, no significant difference was observed in expression of FN in either time intervals (*P*>0.05). Expression of TN in MTA and Propolis groups significantly increased with time (*P*=0.03 and *P*=0.045, respectively). In PRP and GI groups, there was no remarkable difference regarding TN production after 30 days (*P*>0.05).

No recognizable difference was observed among the 7-day samples in expression of FN (*P*=0.235) and TN (*P*=0.903). Significant differences were however present in 30-day specimen in this regard (*P*=0.004 and 0.005, for FN and TN, respectively). The highest and lowest expressions belonged to the MTA and GI, respectively (Tables 1 and 2).

## Discussion

The present animal immunohistochemical study, evaluated the expression of FN and TN as two inactive markers of dentinogenesis in the pulps directly capped with MTA, PRP or Propolis, during different time intervals. The results showed that expression of FN and TN significantly increased with time in MTA group. TN expression also elevated significantly in Propolis group after 30 days. 

The natural regenerative ability of the pulp has already been established. FN and TN are two non-collagenous glycoproteins of ECM, which are expressed during dentinogenesis and can induce odontoblast differentiation [18, 20]. A FN-rich matrix might serve as a reservoir of GFs and signaling molecules for the differentiation of odontoblasts in tertiary dentinogenesis [21] as well as substrates for the adhesion and migration of pulp cells. Such adhesion seems to be significant when it comes to the differentiation of mineralized tissue forming cells [22].

TN can facilitate the adhesion between cells and FN while facilitating the cell migration through the FN-rich matrix [23]. Moreover, TN is associated with the ability of pulp cells to differentiate into HTB forming cells [18]. Piva *et al.* [15] evaluated the expression of FN and TN following DPC with calcium hydroxide in 1-, 7-, 14- and 30-day intervals. The immunohistochemical pattern of FN and TN in the 7-and 14-day intervals was similar to the 1-day samples. Immunostaining for both FN and TN increased on the 30^th^ day. Zarrabi *et al.* [24] evaluated the expression of FN and TN in the human pulp cells capped with MTA and calcium-enriched mixture (CEM) cement, in 2- and 8-week intervals. In the 2-week interval, they reported increased expression of FN and TN beneath both materials; however, the expression of both markers decreased after 8 weeks. In the present study, GI was used as the negative control in the present research, as previous studies have revealed no differentiation of odontoblast-like cells after DPC with GI [25, 26]. 

**Table 1:** Summary of FN Expression after 7 and 30 days [N (%)]

	**7 Days**				**30 Days**			
	Group I	Group II	Group III	Group IV	Group I	Group II	Group III	Group IV
**PRP**	0 (0%)	2 (33.3%)	4 (66.6%)	0 (0%)	0 (0%)	3 (50%)	3 (50%)	0 (0%)
**Propolis**	1 (16.6%)	2 (33.3%)	3 (50%)	0 (0%)	0 (0%)	0 (0%)	5 (83.3%)	1 (16.6%)
**MTA**	0 (0%)	3 (50%)	3 (50%)	0 (0%)	0 (0%)	0 (0%)	4 (66.6%)	2 (33.3%)
**GI**	0 (0%)	3 (50%)	2 (33.3%	1 (16.6%)	2 (33.3%)	3 (50%)	1 (16.6%)	0 (0%)

**Table 2:** Summary of TN Expression after 7 and 30 days [N (%)]

	**7 Days**				**30 Days**			
	Group I	Group II	Group III	Group IV	Group I	Group II	Group III	Group IV
**PRP**	0 (0%)	2 (33.3%)	4 (66.6%)	0 (0%)	0 (0%)	3 (50%)	3 (50%)	0 (0%)
**Propolis**	1 (16.6%)	2 (33.3%)	3 (50%)	0 (0%)	0 (0%)	0 (0%)	5 (83.3%)	1 (16.6%)
**MTA**	0 (0%)	3 (50%)	3 (50%)	0 (0%)	0 (0%)	0 (0%)	4 (66.6%)	2 (33.3%)
**GI**	0 (0%)	3 (50%)	2 (33.3%	1 (16.6%)	2 (33.3%)	3 (50%)	1 (16.6%)	0 (0%)

Propolis can induce the formation of TGF-*β1* which is important for the differentiation of odontoblasts [27] and can synthesize collagen via pulp cells [28]. PRP (a suspension of GFs found in platelets) is an innovation in the medicine and dental science. These GFs play an important role in wound healing and tissue regeneration. Among these GFs, platelet derived growth factor (PDGF) and TGF-*β* have a significant role [29]. Hence, the attempt of the present study was to use PRP and Propolis for pulp capping procedures in order to demonstrate their inductive effects on the expression of FN and TN.

Based on the present study, MTA can form FN- and TN-rich matrix after DPC. MTA has been proved to be effective in triggering HTB formation that may be due to the progressive enhancement of FN and TN synthesis in dental pulp cells. Due to its alkaline pH, MTA can extract GFs from the adjacent dentin. The extracted GFs might potentially influence the expression of TN [30] and are thought to be responsible for stimulating tertiary dentinogenesis [31].

Parolia *et al.* [12] did DPC in human teeth with Propolis, MTA and Dycal and reported that the pulp response with Propolis was similar to MTA. In the present study, although both MTA and Propolis induced higher levels of FN and TN expression in the 30-day interval compared to the 7-day interval, the amount of expressed markers was significantly more in MTA samples. 

Liu *et al.* [14] carried out an immunohistochemical study in order to evaluate the mineralization effect of PRP on human pulp cells and revealed that the newly formed HTB was only visible in the 4- and 8-week samples in the pulp cells adjacent to PRP. They concluded that PRP has good tissue compatibility and can induce formation of hard tissues. In the present study no changes were observed in the expression of FN and TN in the 7- and 30-day intervals. However, in the PRP group, the expression of TN decreased as time progressed, although this was not significant. This decline might be due to the fast degranulation of cells, and rapid release and degradation of GFs in the PRP. It was proposed that the activity of GFs may end as early as 7-10 days [32]. Some authors suggest that the sustained release from PRP gives it some advantages over the other agents [32, 33]. 

The problem with using PRP is the technical sensitivity of the several steps involved in its preparation which increases the risk of preparation errors. The several clinical, laboratory and case reports available on the use of PRP in medicine and dentistry have yielded conflicting results [34, 35]. Accordingly, consistent results cannot be expected unless ideal concentration of platelets and standard PRP preparation techniques are determined.

## Conclusion

Although both Propolis and PRP are acceptable materials for the induction of dentinal bridge formation, MTA still seems to be more effective for this purpose. Similar studies with longer time periods and larger sample sizes are recommended to clarify the quality of the HTB after capping with PRP and Propolis.
